# Supplementary material for: Mechanistic Insights into Dual-Active Liver and Blood-Stage Antiplasmodials
Source: bioRxiv. 2025 Aug 6:2025.08.06.666330. Preprint. [Version 1] doi: 10.1101/2025.08.06.666330 (PMC12340835; doi:10.1101/2025.08.06.666330)
Supplement: Supplement 2 [file NIHPP2025.08.06.666330v1-supplement-2.pdf]

bioRxiv preprint doi: https://doi.org/10.1101/2025.08.06.666330; this version posted August 6, 2025. The copyright holder for this preprint (which was not certified by peer review) is the author/funder, who has granted bioRxiv a license to display the preprint in perpetuity. It is made available under aCC-BY 4.0 International license.

Supplementary Tables

Supplementary Table 1. Compound structures and their anti-plasmodial activity against *P. falciparum* strains Dd2 and 3D7.

Supplementary Table 2. Antiplasmodial activity of selected compounds across a panel of geographically diverse *P. falciparum* strains.

Supplementary Table 3. Single-nucleotide variants (SNVs) identified in clones selected with TCMDC-125075.

Supplementary Table 4. Copy number variations (CNVs) detected in clones following compound selection against TCMDC-125075, TCMDC-124602, TCMDC-141334, TCMDC-140674.

Supplementary Table 5. *In vitro* enzymatic activity of recombinant PfAcAS, HsAcAS, and mutant variants (PfAcAS-T648M and PfAcAS-A597V) in response to TCMDC-125075.

Supplementary Table 6. Single-nucleotide variants (SNVs) identified in clones selected with TCMDC-124602.

Supplementary Table 7. *In vitro* inhibition of *P. falciparum* cytoplasmic isoleucyl-tRNA synthetase (PfcIRS) by TCMDC-124602 using recombinant protein assays.

Supplementary Table 8. Single-nucleotide variants (SNVs) identified in clones selected with TCMDC-141334.

Supplementary Table 9. Single-nucleotide variants (SNVs) identified in clones selected with TCMDC-140674.

Supplementary Table 10. Composition of the barcoded AReBar pool at the start (Day 0) and end (Day 14) of the experiment, either untreated or in the presence of compound TCMDC-134122.

bioRxiv preprint doi: https://doi.org/10.1101/2025.08.06.666330; this version posted August 6, 2025. The copyright holder for this preprint (which was not certified by peer review) is the author/funder, who has granted bioRxiv a license to display the preprint in perpetuity. It is made available under aCC-BY 4.0 International license.

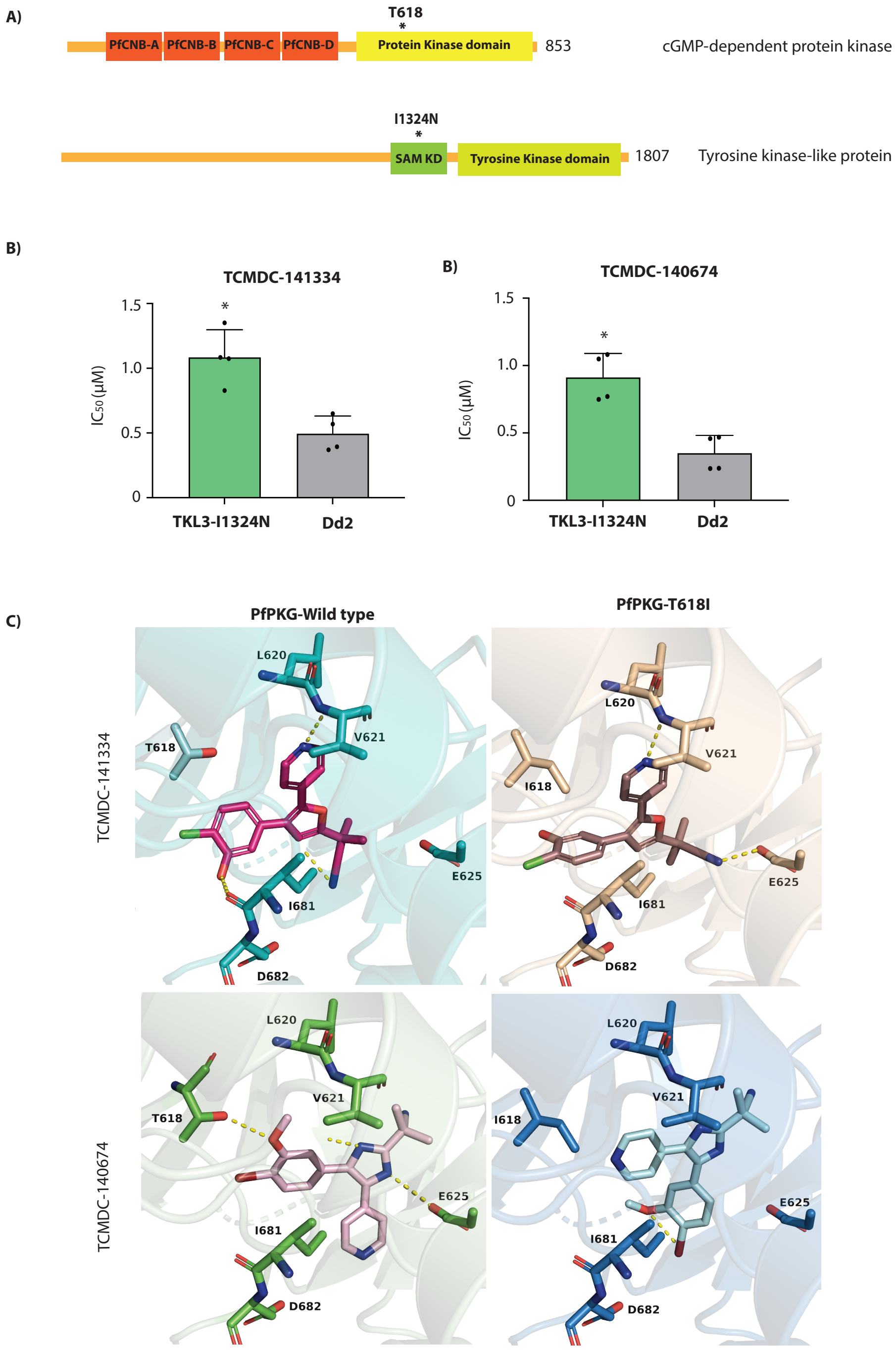

Supplementary Figure 1. TCMDC-141334 and TCMDC-140674 target *P. falciparum* cGMP-dependent protein kinase (PfPKG). (A) Schematic representation of the domain architecture of PfPKG and PfTKL3. **(B)** CRISPR-edited parasite lines carrying the *Pf*TKL3 **I**1342N mutation exhibited reduced sensitivity to both TCMDC-141334 and TCMDC-140674. The Dd2 parental line was included as a reference. Each dot represents a biological replicate (n = 4); bars indicate mean ± SD and statistical significance determined by Mann-Whitney *U* tests (*p<0.05, **p<0.01). **(C)** Molecular docking models showing the binding of TCMDC-141334 and TCMDC-140674 to both wild-type PfPKG and the PfPKG-T618I mutant.
